# The Use of the Internal Transcribed Spacer Region for Phylogenetic Analysis of the Microsporidian Parasite *Enterocytozoon hepatopenaei* Infecting Whiteleg Shrimp (*Penaeus vannamei*) and for the Development of a Nested PCR as Its Diagnostic Tool

**DOI:** 10.4014/jmb.2401.01010

**Published:** 2024-02-27

**Authors:** Ju Hee Lee, Hye Jin Jeon, Sangsu Seo, Chorong Lee, Bumkeun Kim, Dong-Mi Kwak, Man Hee Rhee, Patharapol Piamsomboon, Yani Lestari Nuraini, Chang Uook Je, Seon Young Park, Ji Hyung Kim, Jee Eun Han

**Affiliations:** 1 Laboratory of Aquatic Biomedicine, College of Veterinary Medicine, Kyungpook National University, Daegu 41566, Republic of Korea; 2 Department of Veterinary Medicine, Faculty of Veterinary Science, Chulalongkorn University, Bangkok, Thailand; 3 Veterinary Medical Aquatic Animal Research Center of Excellence, Chulalongkorn University, Bangkok, Thailand; 4 Fish Health and Environmental Laboratory, Brackishwater Aquaculture Development Center, Situbondo, Indonesia; 5 Ministry of Agriculture, Food and Rural Affairs, Sejong 30110, Republic of Korea; 6 Division of Animal and Dairy Sciences, College of Agriculture and Life Science, Chungnam National University, Daejeon 34134, Republic of Korea; 7 Department of Food Science and Biotechnology, Gachon University, Seongnam 13120, Republic of Korea; 8 Institute for Veterinary Biomedical Science, Kyungpook National University, Daegu 41566, Republic of Korea

**Keywords:** *Enterocytozoon hepatopenaei*, internal transcribed spacer, microsporidia, phylogeny, polymerase chain reaction, shrimp

## Abstract

The increasing economic losses associated with growth retardation caused by *Enterocytozoon hepatopenaei* (EHP), a microsporidian parasite infecting penaeid shrimp, require effective monitoring. The internal transcribed spacer (ITS)-1 region, the non-coding region of ribosomal clusters between 18S and 5.8S rRNA genes, is widely used in phylogenetic studies due to its high variability. In this study, the ITS-1 region sequence (~600-bp) of EHP was first identified, and primers for a polymerase chain reaction (PCR) assay targeting that sequence were designed. A newly developed nested-PCR method successfully detected the EHP in various shrimp (*Penaeus vannamei* and *P. monodon*) and related samples, including water and feces collected from Indonesia, Thailand, South Korea, India, and Malaysia. The primers did not cross-react with other hosts and pathogens, and this PCR assay is more sensitive than existing PCR detection methods targeting the small subunit ribosomal RNA (SSU rRNA) and spore wall protein (SWP) genes. Phylogenetic analysis based on the ITS-1 sequences indicated that the Indonesian strain was distinct (86.2% nucleotide sequence identity) from other strains collected from Thailand and South Korea, and also showed the internal diversity among Thailand (*N* = 7, divided into four branches) and South Korean (*N* = 5, divided into two branches) samples. The results revealed the ability of the ITS-1 region to determine the genetic diversity of EHP from different geographical origins.

## Introduction

*Enterocytozoon hepatopenaei* (EHP), the causative agent of hepatopancreatic microsporidiosis, is an intracellular parasite infecting the cytoplasm of hepatopancreatic tubule epithelial cells of the shrimp. It was first reported in a population with slow growth among *Penaeus monodon* cultured in Thailand [1] and was later named EHP based on its histopathology, ultra-structural features, and small subunit ribosomal RNA (SSU rRNA) gene sequence [2]. In fact, the mortality associated with EHP is not that high; however, growth retardation is causing great economic losses to the shrimp aquaculture industry. Furthermore, EHP has been overlooked for some time and widely spread in various Asian countries because shrimps died before EHP symptoms appeared due to the prevalence of acute hepatopancreatic necrosis disease (AHPND), which has a high mortality rate [3]. Although EHP is not directly associated with immune responses, it weakens the defense system by damaging the hepatopancreas and midgut, the organs that store nutrients and energy needed to support growth and metabolic function, increasing the susceptibility to other pathogens, such as *Vibrio* and acute hepatopancreatic necrosis disease (AHPND) [4]. Moreover, no specific clinical signs have been reported in EHP-infected shrimps, thus making the monitoring of EHP infection and controlling its spread difficult [5]. Therefore, a rapid and simple detection method should be urgently developed for EHP infection in the global shrimp aquaculture industry.

To date, several diagnostic methods for EHP detection have been reported using the SSU rRNA gene and spore wall protein (SWP) gene [5-8]. Although these genes have been proven suitable targets for the molecular diagnosis of EHP infection, the potential internal genetic diversity of EHP among the different geographical origins and host species could not be evaluated due to the high nucleotide sequence similarity among closely related species.

Internal transcribed spacer (ITS), another ribosomal cluster, is a non-coding region located between the SSU and large subunit ribosomal DNA coding region, and is classified into ITS-1 and ITS-2 based on the 5.8S rDNA coding region [9]. Several studies, including microsporidia studies, reported that the ITS-1 region is widely used in phylogenetic studies due to its high variability [10, 11]. In *Enterocytozoon bieneusi*, one of the pathogenic microsporidia genetically related to EHP, the ITS-1 region was frequently used for its phylogenetic analyses and diagnoses [12, 13]. However, the exact nucleotide sequence of the ITS-1 region of EHP has not yet been identified. In this study, the ITS-1 region sequence of EHP was first identified using next-generation sequencing analyses, and a PCR assay was developed based on the ITS-1 region sequence. Furthermore, the discrimination ability of the newly developed PCR assay was evaluated to determine the potential genetic variation of shrimp-infected EHP from different geographical origins (including Indonesia, Thailand, and South Korea). To the best of our knowledge, this study first identified the ITS-1 region of EHP and its application for molecular diagnostic use.

## Materials and Methods

### EHP Samples

A total of 25 EHP-positive samples were collected in this study: 1) one fecal sample of *Penaeus vannamei* (*P. vannamei*) showing slow growth and white feces syndrome from Indonesia, 2) eight hepatopancreatic samples of the EHP-positive *P. vannamei* collected from Thailand, 3) two EHP-positive culture-pond water samples and 11 hepatopancreatic samples of the EHP-positive *P. vannamei* collected from South Korea, 4) two hepatopancreatic samples of the EHP-positive *P. monodon* collected from India, and 5) one hepatopancreatic samples of the EHP-positive *P. monodon* collected from Malaysia (Table 1). The presence of EHP in all collected samples was preliminarily confirmed by polymerase chain reaction (PCR) analyses using primer sets described by Tang *et al*.(2015) and Jaroenlak *et al*. (2016) (Table 2). All collected samples were stored at −80°C until use.

### EHP Genome Sequencing

To identify the ITS-1 region sequence of EHP, two EHP-infected shrimp samples (sample no. 1: EHP-ID16, Indonesia; sample no. 2: 21-079B1, Republic of Korea) were used in this study. To obtain the genome sequence of the two EHP strains, 1) the extracted total genomic DNA of the EHP-ID16 was sequenced using the Hiseq4000 sequencing platform (Illumina, USA), and assembled using SPAdes (v3.12.0) [14] at Macrogen Inc. (Republic of Korea); 2) the extracted total genomic DNA of the EHP 21-079B1 was sequenced using the HiSeqXten sequencing platform (Illumina), and assembled using Platanus-allee (v2.2.2) [15] at Macrogen Inc. Filtered Illumina paired-end reads of EHP-ID16 (25,062,696,326-bp, 260,481,872 reads) and 21-079B1 (8,519,044,010-bp, 56,417,510 reads) were obtained, and presumptive EHP-associated sequences were retrieved by mapping with other available EHP genomes (strain TH1, MNPJ00000000.1) [16] in the GenBank database. Among the two sequenced EHP genomes, the finally obtained contigs of the sample EHP-ID16 were preferentially used for further analyses in this study.

### Obtaining the ITS-1 Region Sequence from the EHP Genome

Using the obtained presumptive EHP-associated contigs of the EHP-ID16 sample, 18S and 5.8S rRNA gene-containing contigs were manually searched by BLAST against those other available sequences from EHP, *i.e.*, *E. bieneusi* and the genus *Nucleospora* spp. in the GenBank database. Then, a pair of PCR primers (forward: ITS1-1F; reverse: ITS1-1R) (Table 2) were designed using 18S and 5.8S rRNA-containing contigs of the EHP-ID16, and a PCR assay was performed using the genomic DNA of EHP strains EHP-ID16 and 21-079B1 (Table 1) under the following cycling conditions: initial denaturation at 95°C for 5 min; followed by 35 cycles at 95°C for 40 s, 58°C for 40 s, and 72°C for 40 s; and a final extension at 72°C for 5 min. The amplified PCR products were sequenced using ITS1-1F/1R primers at Macrogen Inc., and the exact nucleotide sequence of the ITS-1 region of EHP-ID16 and 21-079B1 samples was finally confirmed.

### Development of the ITS-1-Based Nested-PCR Assay

Before performing the PCR assay, the total genomic DNA of the collected EHP-positive samples (*N* = 25, Table 1) was extracted using the DNeasy® Blood & Tissue Kit (Qiagen Korea Ltd., Republic of Korea) following the manufacturer’s instructions. To develop the ITS-1-based nested-PCR assay, a first-step PCR was performed with the ITS1-1F/1R primers under the above-mentioned cycling conditions using the South Korean EHP strain 21-079B1 (Table 1). Then, to increase sensitivity, a second-step (nested) PCR was carried out with ITS1-2F/2R internal primers (Table 2). One microliter of PCR products from the first-step reaction was used as the template DNA. Amplification was performed under the following cycling conditions: initial denaturation at 95°C for 5 min; followed by 20 cycles at 95°C for 30 s, 62°C for 30 s, and 72°C for 30 s; and a final extension at 72°C for 5 min.

To confirm the specificity of the newly designed nested-PCR primers, the potential amplification of other host-or pathogen-associated DNAs was separately examined using DNAs of healthy shrimp (*P. vannamei*, *N* = 1; *P. monodon*, *N* = 1), crab (*Macrophthalmus japonicus*, *N* = 1), cuttlefish (*Sepia officinalis*, *N* = 1), infectious hypodermal and hematopoietic necrosis (IHHNV; infected tissue DNA, *N* = 1), white spot syndrome virus (WSSV; infected tissue DNA, *N* = 1), AHPND-associated *Vibrio parahaemolyticus* (bacteria DNA, *N* = 1), non-AHPND *V. parahaemolyticus* (bacteria DNA, *N* = 1), *V. harveyi* (bacteria DNA, *N* = 2), and other closely related microsporidia, *E. bieneusi* (infected tissue DNA, *N* = 1).

To confirm the sensitivity of the newly designed nested-PCR primers, DNAs (261.3 ng/μl) from the EHP strain 21-079B1 were serially diluted (a 10-fold dilution, 10^−1^ to 10^−5^) and amplified. Moreover, the primer sets (ITS1-1F/ 1R and ITS1-2F/2R) were compared with the previously described primer sets (SWP-1F/1R, SWP-2F/2R, and 510-F/510-R) described by Jaroenlak *et al*. (2016) and Tang *et al*. (2015) (Table 2).

### Phylogenetic Analysis of the EHP ITS-1 Region

The representative positive PCR amplicons in the EHP ITS-1 region of Indonesian (*N* = 1, EHP-ID16), Thailand (*N* = 6, 22-044A2, 22-044A6, 22-044A8, 22-044A12, 22-044A13, and 22-044A14), and South Korean samples (*N* = 5, 21-079B1, 21-044B, 21-084B3, 21-064B3, and 21-061B) were sequenced and used for further phylogenetic analysis. The obtained nucleotide sequences of the EHP ITS-1 region were directly compared with those of the EHP-ID16 and 21-079B1 samples using Geneious Prime (ver. 2023; https://www.geneious.com). For phylogenetic analyses, the ITS-1 datasets of nucleotide sequences from 12 sequenced representative EHP isolates in this study were combined with those obtained from TH1 and aligned using ClustalX (ver. 2.1) [17] and BioEdit Sequence Alignment Editor (ver. 7.1.0.3) [18]. A maximum-likelihood phylogenetic tree was constructed using the Jukes–Cantor model and 1,000 bootstrap replicates using MEGA X (ver. 10.0) [19].

## Results

### EHP-ID16 and 21-079B1 EHP Genome

In this study, two EHP strains infecting *P. vannamei* from different geographical origins were sequenced: 1) EHP-ID16 from Indonesia collected in 2016, and 2) 21-079B1 from South Korea collected in 2021. The presumptive EHP-associated contigs were retrieved by mapping with another available EHP strain TH1 genome (MNPJ00000000.1). Using the mapped sequences (3,004,048,835-bp, 31,214,630 reads) of EHP-ID16 against the TH1 genome, a total of 162 EHP-associated contigs were obtained, and its overall draft genome was estimated to be 2,825,971-bp in size with 25.5% G + C content (924.0 × coverage). In the EHP strain 21-079B1, a total of 86 EHP-associated contigs were obtained from mapped sequences (2,111,211,001-bp, 14,025,866 reads), and its overall draft genome was estimated to be 2,896,287-bp in size with 25.3% G + C content (728.9 × coverage). Sequenced genomes of strains EHP-ID16 and 21-079B1 in this study were deposited at the GenBank database under accession numbers QTJQ00000000.1 and JARHUL000000000.1, respectively.

### ITS-1 Sequence of EHP

To identify the ITS-1 region of EHP, the finally obtained contigs of the strain EHP-ID16 were preferentially used for further analyses. Among the obtained 162 EHP-associated contigs of strain EHP-ID16, each of the contigs possessing 18S rRNA and 5.8S rRNA was finally screened: the presence of 18S rRNA and 5.8S rRNA was detected at contig_63 (QTJQ01000063.1) and contig_64 (QTJQ01000064.1), respectively. The newly identified 18S rRNA and 5.8S rRNA of EHP were mostly similar to those from EHP (98.9%–99.9%) and *Nucleospora* sp. (79.1%–80.0%) by the NCBI BLAST search, respectively.

Therefore, the obtained two rRNA-containing contigs were used to determine the nucleotide sequence of the ITS-1 region, and the intermediate sequence region between 18S and 5.8S rRNA was considered for the ITS-1 region of EHP. Simultaneously, a pair of PCR primers (ITS1-1F, targeting the downstream of 18S rRNA, and ITS1-1R, targeting the upstream of 5.8S rRNA) was designed, and the PCR and sequencing analyses were conducted using EHP strains EHP-ID16 and 21-079B1 to cross-check the accuracy of the obtained ITS-1 sequence of EHP.

The newly designed PCR primers (ITS1-1F/1R) successfully amplified ~600-bp amplicon of EHP-ID16, and its sequencing analysis showed 100% nucleotide sequence identity to the two 18S and 5.8S rRNA-containing contigs obtained by the NGS analysis in this study. Therefore, the sequenced ITS-1 region of EHP-ID16 was finally estimated to be 456-bp in length. Although the ITS1-1F and ITS1-1R primers also successfully amplified the expected region of the strain 21-079B1 containing 100% identical sequences of 18S and 5.8S rRNAs of EHP-ID16, its ITS-1 was estimated to be 438-bp in length. Direct sequence comparison of the predicted ITS-1 region between strains EHP-ID16 and 21-079B1 revealed 86.2% nucleotide identity, thus determining the potential internal diversity of the ITS-1 region of EHP strains from different geographical origins. However, the two obtained sequences of the EHP ITS-1 region demonstrated no relevant homology to any reported microsporidia-related sequences in the GenBank database. The finally obtained 438-bp ITS-1 region sequence of the EHP strain 21-079B1 was further used for further PCR primer designation in this study and was deposited at the GenBank database under the accession number ON015652.

### Development of ITS-1-Based Nested-PCR Assay

For diagnostic purposes, a nested-PCR assay targeting the EHP ITS-1 region was performed. The first-step PCR using EHP-infected shrimp samples (*N* = 22), water (*N* = 2), and feces (*N* = 1) as templates generated ~600-bp amplicons, except for one water sample (21-044A2). However, the nested-PCR assay showed improved detection capability against EHP: ~400-bp amplicons were obtained from all samples used in the first-step PCR including the water sample (21-044A2), which did not show an amplicon during the first-step PCR (Table 1).

**Fig. 1:**
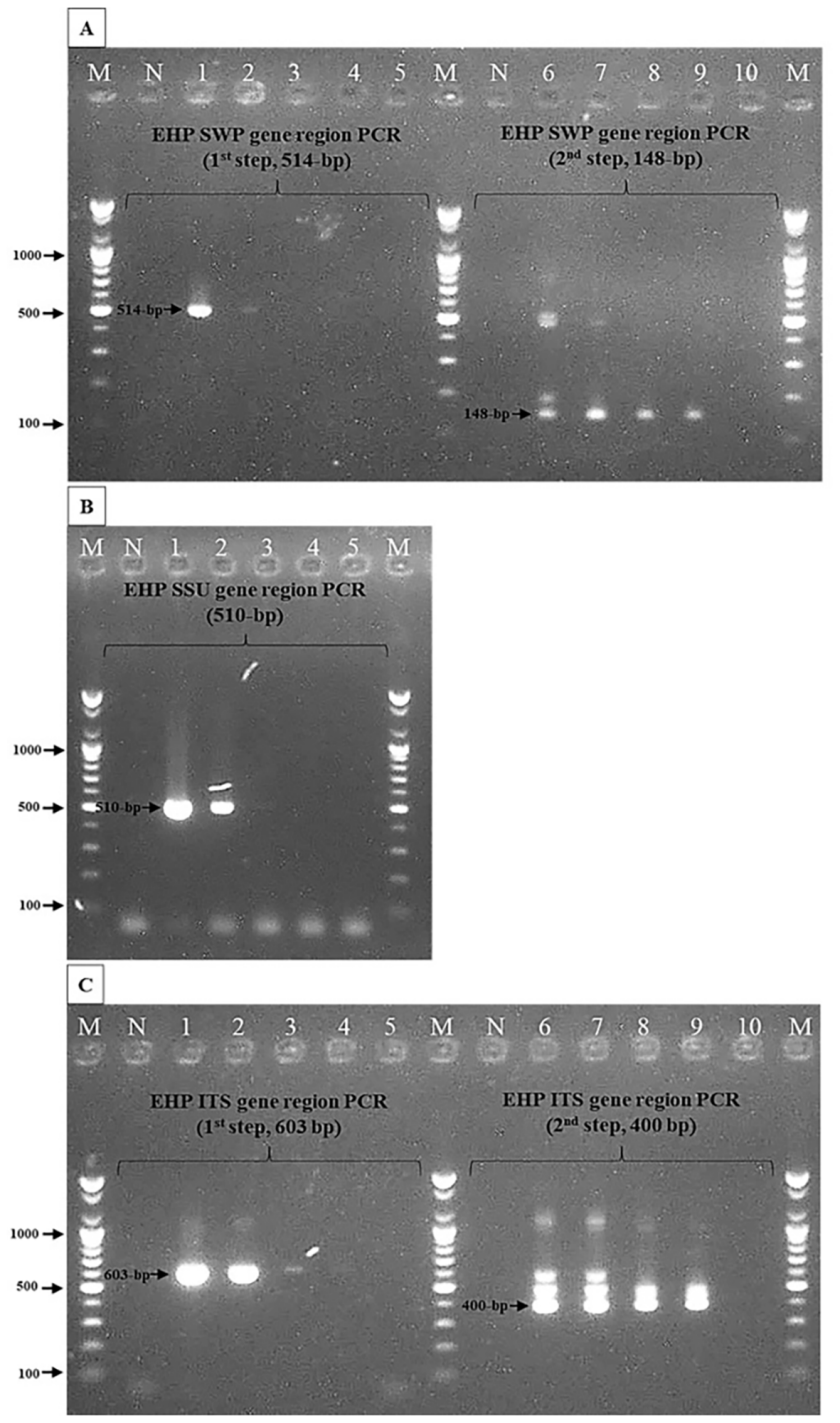
Sensitivity comparison between the SSU, SWP, and ITS-1 PCR assays determined by a 10-fold dilution (from 10^−1^ to 10^−5^) of EHP-positive DNA (21-079B1). Lane M: 100 bp DNA ladder; Lane N: negative control (DEPCwater). (**A**) SWP-PCR first-step (514 bp) and nested-step (148 bp) PCR result. Lanes 1-5: 10^−1^ to 10^−5^ dilution (first step); Lanes 6-10: 10^−1^ to 10^−5^ dilution (nested step). (**B**) SSU-PCR (510 bp) result. Lanes 1-5: 10^−1^ to 10^−5^ dilution. (**C**) ITS-1-PCR first-step (603 bp) and nested-step (400 bp) PCR result. Lanes 1-5: 10^−1^ to 10^−5^ dilution (first step); Lanes 6-10: 10^−1^ to 10^−5^ dilution (nested step).

In the specificity test, no samples showed bands from the DNAs of healthy shrimp (*P. vannamei*, *N* = 1; *P. monodon*, *N* = 1), crab (*N* = 1), and cuttlefish (*N* = 1), thus confirming that the newly developed ITS1-1F/1R and 2F/2R primers did not react to the host genome (data not shown). Moreover, the primers did not cross-react with other pathogens, including *E. bieneusi*, which is closely related to EHP, and other shrimp pathogens such as IHHNV, WSSV, AHPND-associated *V. parahaemolyticus*, non-AHPND *V. parahaemolyticus*, and *V. harveyi* (data not shown). In the sensitivity test, the nested-step PCR using primer sets (ITS1-1F/1R and ITS1-2F/2R) showed stronger amplicons compared to the PCR using primer sets described by Tang *et al*. (2015) and Jaroenlak *et al*. (2016). By examining serial dilutions of DNAs from EHP-infected shrimp, the nested step of ITS-1-PCR assay showed a 10-fold increase and similar sensitivity to PCR assays using 510-F/R [3] and SWP-1F/1R and 2F/2R primers [6], respectively (Fig. 1).

### Phylogenetic Analysis of the ITS-1 Region of EHPs from Different Geographical Origins

To compare the ITS-1 region of EHPs from different geographical origins, several representative EHP-positive samples collected from Indonesia and South Korea (EHP-ID16 and 21-079B1) were selected and sequenced for further analyses. Obtained sequences of the EHP ITS-1 region were almost identical (>99% identity) to that from the EHP strain 21-079B1, except for the strain EHP-ID16, which showed 86.2% sequence identity with two internal gaps (Fig. S1).

**Fig. 2:**
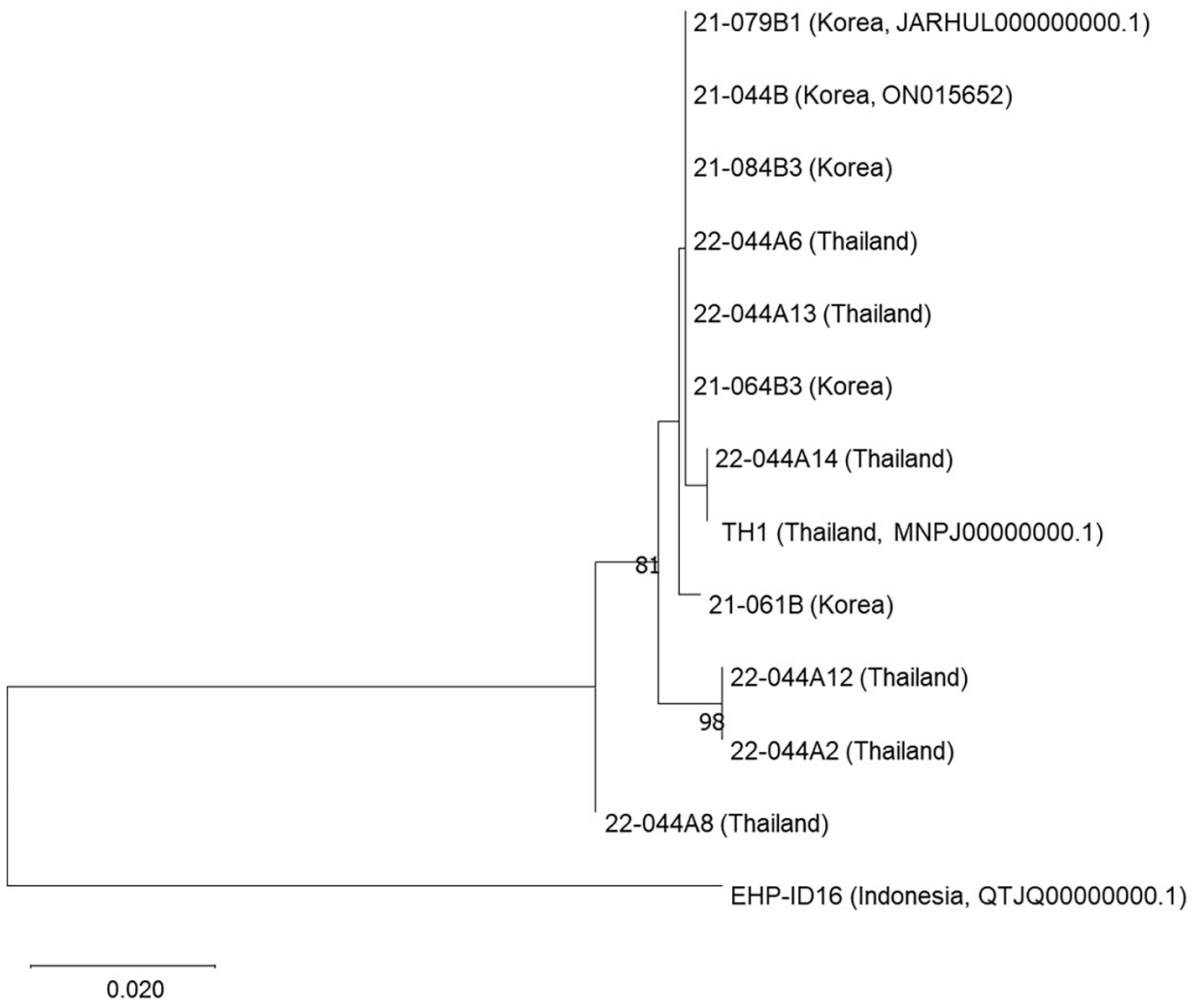
Phylogenetic analyses of the representative ITS-1 region of EHPs obtained in this study. Maximumlikelihood trees were reconstructed based on the obtained representative sequences of the EHP-positive samples and GenBank database. The numbers at the branches indicate bootstrap values obtained with 1,000 replicates. The scale bar represents 0.1 nucleotide substitution per site.

To evaluate the potential internal diversity of EHPs from different geographical origins, phylogenetic analysis was performed using the nucleotide dataset of 13 ITS-1 regions consisting of EHP samples collected from Indonesia (*N* = 1), Thailand (*N* = 7; six from this study and one from GenBank, accession no. MNPJ00000000.1), and South Korea (*N* = 5). The resultant phylogeny indicated that the strain EHP-ID16 was clearly differentiated from other EHPs collected from Thailand and South Korea and also revealed the internal diversity of EHPs among the Thai and South Korean samples (Fig. 2).

## Discussion

Recently, EHP outbreaks on shrimp farms have been widely reported in Venezuela and Asian countries including China, Vietnam, India, Indonesia, Malaysia, Thailand, and South Korea [4, 20-23]. The increasing economic loss associated with EHP in shrimp farms makes it necessary to develop effective monitoring and diagnostic methods to improve its management. Furthermore, a more specific target gene sequence showing high variability should be identified to determine its transmission route. For EHP diagnosis, the SSU rRNA gene sequence [2], and the SWP gene sequence have been widely used [6]. These methods using the SSU rRNA gene and the SWP gene sequence produce rapid and sensitive diagnostic results; however, seeing the genetic diversity among related species or genera and origins is difficult. As the SSU rRNA gene sequence is highly conserved, a little barcode gap is observed in DNA barcoding, which classifies species by DNA sequence differences; therefore, the classification based on the SSU rRNA gene in species or genus level is poor [24-25]. This characteristic is also associated with the binding of primers to the DNA of closely related microsporidia in PCR detection targeting the SSU rRNA gene to cause false positives [6]. Therefore, a new criterion should be considered to distinguish EHP from other microsporidia and determine the potential genetic variation from different geographical origins.

ITS-1, located between 18S and 5.8S rDNA coding regions, is highly variable between related species compared to the SSU rRNA gene [26]. Due to its high variability, ITS-1 has been suggested as a universal DNA barcode marker for fungi [24], and this sequence is suitable for analyzing the organism groups that are closely related and emerged recently [11]. Moreover, for diagnostic purposes, the ITS-1 region sequence is suitable as a PCR target as the sequence exists between the conserved sequences, 18S, and 5.8S rRNA, making it easy to design primers [26], and each cell has several rRNA gene copies to satisfy the demand for protein synthesis, thereby identifying a significant number of PCR targets [26, 27].

In this study, the ITS-1 sequence was firstly identified from the EHP-infected shrimp and a PCR assay was developed based on this sequence. Based on the result of the PCR assay, a newly developed PCR detection method targeting the ITS-1 region was useful for EHP diagnosis, which generated amplicons from EHP-infected shrimp, water, and feces samples, and the primers were specific to EHP that nested PCR did not cross-react with the genomic DNA of other aquatic organisms other than shrimp and with DNA of related pathogens. Furthermore, the sensitivity test revealed that the nested step of ITS-1 PCR had stronger bands compared with the first step, indicating that this method would help detect low EHP levels in samples. Moreover, compared with existing PCR methods, the first step of ITS-1 PCR was more sensitive than SWP-PCR and SSU-PCR in the first step and similar to SWP-PCR in the nested step. This would be helpful to save time by performing the first step alone during mass inspection.

Furthermore, the ITS-1 region-based resultant phylogeny showed that the Indonesian EHP was distinct from other EHPs collected in this study, and also revealed the internal diversity of EHPs among the Thailand and South Korean samples. These results indicate the potential availability of the ITS-1 region of EHP to discriminate the internal genetic diversity of the pathogen from different geographical origins.

In conclusion, this new, sensitive, and specific PCR detection method can be a valuable means of monitoring and diagnosing EHP in shrimp samples as this method is available to detect EHP from samples with low EHP infection levels and to estimate inflow routes and EHP variation. The ITS-1 region sequence first identified in this study also can be used in a further study classifying microsporidia and EHP subtypes.

## Supplemental Materials

Supplementary data for this paper are available on-line only at http://jmb.or.kr.



## Figures and Tables

**Table 1:** Sample information and PCR results of *Enterocytozoon hepatopenaei* (EHP) in this study.

Sample ID	Year/Month	Country/Province/area	Aquafarm	Sample types (numbers)	EHP PCR assays		
					SWP	SSU	ITS
19-003C (EHP-ID16)	2016/01	Indonesia/Java/Situbondo	Farm 1	Feces (*N* = 1)	+	+	+
21-044A1	2021/06	South Korea/Incheon/Ganghwa-gun	Farm 2	Water (*N* = 1)	+	-	+
21-044A2	2021/06	South Korea/Incheon/Ganghwa-gun	Farm 2	Water (*N* = 1)	+	+	+
21-044B2	2021/06	South Korea/Incheon/Ganghwa-gun	Farm 2	*P. vannamei* (*N* = 3)	+	+	+
21-079B1	2021/08	South Korea/Incheon/Ganghwa-gun	Farm 2	*P. vannamei* (*N* = 3)	+	+	+
21-061B	2021/08	South Korea/Jellolabuk-do/Gochang-gun	Farm 3	*P. vannamei* (*N* = 3)	+	+	+
21-064B3	2021/08	South Korea/Chungcheongnam-do/Taean-gun	Farm 4	*P. vannamei* (*N* = 3)	+	+	+
21-084B3	2021/09	South Korea/Chungcheongnam-do/Taean-gun	Farm 4	*P. vannamei* (*N* = 3)	+	+	+
21-084 B3-S1	2021/09	South Korea/Chungcheongnam-do/Taean-gun	Farm 4	*P. vannamei* (*N* = 1)	+	+	+
21-084 B3-S2	2021/09	South Korea/Chungcheongnam-do/Taean-gun	Farm 4	*P. vannamei* (*N* = 1)	+	+	+
21-084 B3-S3	2021/09	South Korea/Chungcheongnam-do/Taean-gun	Farm 4	*P. vannamei* (*N* = 1)	+	+	+
21-084 B3-S4	2021/09	South Korea/Chungcheongnam-do/Taean-gun	Farm 4	*P. vannamei* (*N* = 1)	+	+	+
21-084 B3-S5	2021/09	South Korea/Chungcheongnam-do/Taean-gun	Farm 4	*P. vannamei* (*N* = 1)	+	+	+
21-084 B3-S6	2021/09	South Korea/Chungcheongnam-do/Taean-gun	Farm 4	*P. vannamei* (*N* = 1)	+	+	+
22-044A2	2022/10	Thailand	Farm 5	*P. vannamei* (*N* = 10)	+	+	+
22-044A6	2022/10	Thailand	Farm 6	*P. vannamei* (*N* = 10)	+	+	+
22-044A8	2022/10	Thailand	Farm 7	*P. vannamei* (*N* = 10)	+	+	+
22-044A10	2022/10	Thailand	Farm 8	*P. vannamei* (*N* = 10)	+	-	+
22-044A12	2022/10	Thailand	Farm 9	*P. vannamei* (*N* = 10)	+	+	+
22-044A13	2022/10	Thailand	Farm 10	*P. vannamei* (*N* = 10)	+	+	+
22-044A14	2022/10	Thailand	Farm 11	*P. vannamei* (*N* = 10)	+	+	+
22-044A15	2022/10	Thailand	Farm 12	*P. vannamei* (*N* = 10)	+	+	+
23-026A6-2-INDIA	2022/11	India	Farm 13	*P. monodon* (*N* = 10)	+	+	+
23-026A9-2-INDIA	2022/11	India	Farm 14	*P. monodon* (*N* = 10)	+	+	+
23-026A5-1-MAL	2023/02	Malaysia	Farm 15	*P. monodon* (*N* = 10)	+	+	+

+: Positive, -: Negative

**Table 2:** Primer sequences for the EHP PCR assay used in this study.

Primer	Sequence 5' to 3'	Amplicon size (bp)	Description	Reference
SWP-1F	TTGCAGAGTGTTGTTAAGGGTTT	514	EHP detection targeting SWP	[6]
SWP-1R	CACGATGTGTCTTTGCAATTTTC			
SWP-2F	TTGGCGGCACAATTCTCAAACA	148		
SWP-2R	GCTGTTTGTCTCCAACTGTATTTGA			
510-F	GCCTGAGAGATGGCTCCCACGT	510	EHP detection targeting SSU	[3]
510-R	GCGTACTATCCCCAGAGCCCGA			
ITS1-1F	CGCCCGTCACTATTTCAGAT	603	EHP detection targeting ITS-1	In this study
ITS1-1R	TACGTTCGTCATCGCTGCTA			
ITS1-2F	GAACCTGCTGTGGGATCATT	400		
ITS1-2R	AATTTTTGCTTGGCTCATTCT			
