# Supplementary material for: The Use of the Internal Transcribed Spacer Region for Phylogenetic Analysis of the Microsporidian Parasite Enterocytozoon hepatopenaei Infecting Whiteleg Shrimp (Penaeus vannamei) and for the Development of a Nested PCR as Its Diagnostic Tool
Source: J Microbiol Biotechnol. 2024 Feb 27;34(5):1146–53. doi: 10.4014/jmb.2401.01010 (PMC11180916; doi:10.4014/jmb.2401.01010)
Supplement: Supplementary file 1 [file jmb-34-5-1146-supple.pdf]

J. Microbiol. Biotechnol. 2024. 34(5): 1146–1153

https://doi.org/10.4014/jmb.2401.01010

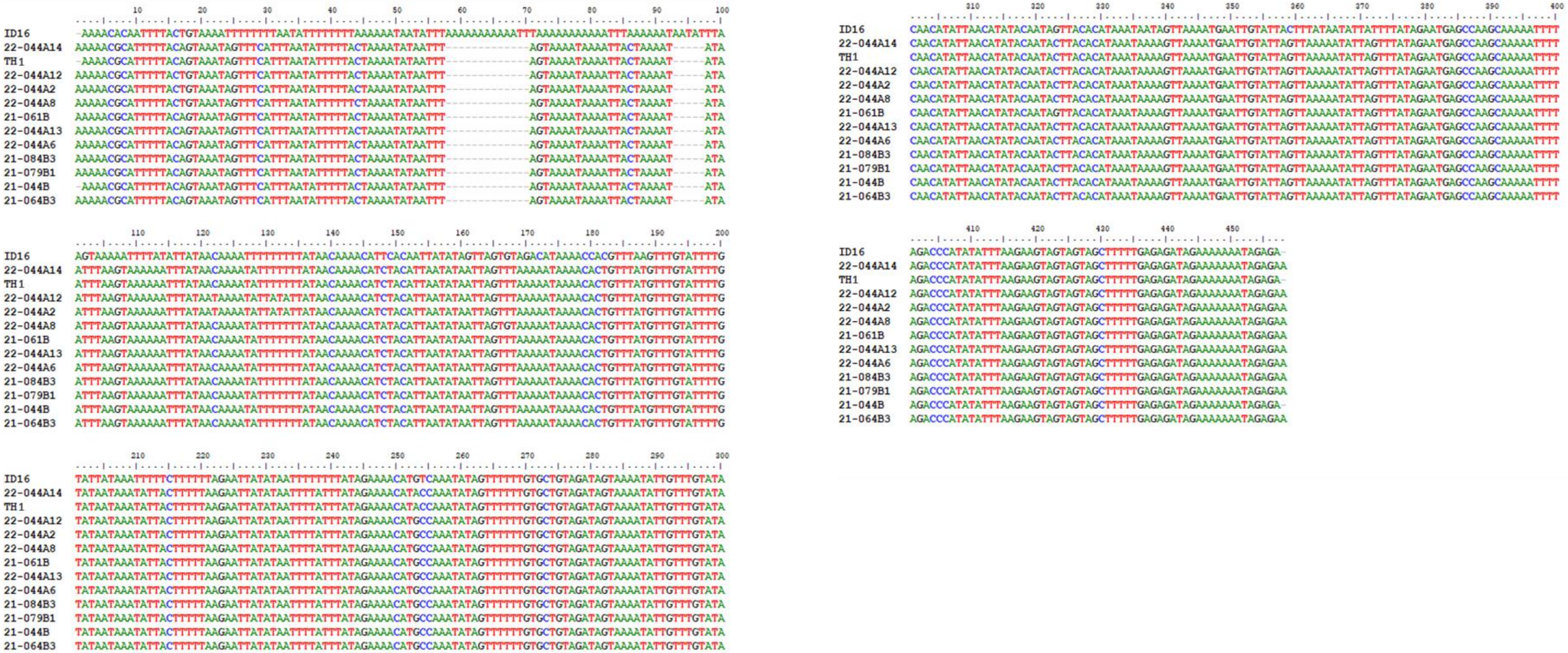

Supplementary Fig. S1. Multiple alignments of the EHP ITS-1 region nucleotide sequences from different geographical origins used in this study.
